# An innovative pay-for-performance (P4P) strategy for improving malaria management in rural Kenya: protocol for a cluster randomized controlled trial

**DOI:** 10.1186/1748-5908-8-48

**Published:** 2013-05-08

**Authors:** Diana Menya, John Logedi, Imran Manji, Janice Armstrong, Brian Neelon, Wendy Prudhomme O’Meara

**Affiliations:** 1 Department of Epidemiology and Nutrition, Moi University School of Public Health, College of Health Sciences, Nandi Road, Eldoret, Kenya; 2 Division of Malaria Control, Ministry of Public Health and Sanitation, Kenyatta Hospital, Nairobi, Kenya; 3 Academic Model Providing Access to Healthcare, Eldoret, Kenya; 4 Department of Family Medicine, Moi University School of Medicine, College of Health Sciences, Nandi Road, Eldoret, Kenya; 5 Division of Infectious Diseases, Duke University School of Medicine, Hanes House, Trent Drive, Durham, NC, USA; 6 Duke Global Health Institute, Trent Hall, Trent Drive, Durham, NC, USA

## Abstract

**Background:**

In high-resource settings, ‘pay-for-performance’ (P4P) programs have generated interest as a potential mechanism to improve health service delivery and accountability. However, there has been little or no experimental evidence to guide the development or assess the effectiveness of P4P incentive programs in developing countries. In the developing world, P4P programs are likely to rely, at least initially, on external funding from donors. Under these circumstances, the sustainability of such programs is in doubt and needs assessment.

**Methods/design:**

We describe a cluster-randomized controlled trial underway in 18 health centers in western Kenya that is testing an innovative incentive strategy to improve management of an epidemiologically and economically important problem—diagnosis and treatment of malaria. The incentive scheme in this trial promotes adherence to Ministry of Health guidelines for laboratory confirmation of malaria before treatment, a priority area for the Ministry of Health. There are three important innovations that are unique to this study among those from other resource-constrained settings: the behavior being incentivized is quality of care rather than volume of service delivery; the incentives are applied at the facility-level rather than the individual level, thus benefiting facility infrastructure and performance overall; and the incentives are designed to be budget-neutral if effective.

**Discussion:**

Linking appropriate case management for malaria to financial incentives has the potential to improve patient care and reduce wastage of expensive antimalarials. In our study facilities, on average only 25% of reported malaria cases were confirmed by laboratory diagnosis prior to the intervention, and the total treatment courses of antimalarials dispensed did not correspond to the number of cases reported. This study will demonstrate whether facility rather than individual incentives are compelling enough to improve case management, and whether these incentives lead to offsetting cost-savings as a result of reduced drug consumption.

**Trial registration:**

ClinicalTrials.gov Registration Number NCT01809873

## Background

Performance-based incentives, also called pay for performance (P4P), have been implemented extensively in the developed world over the last decade [1-4]. More recently, P4P incentive programs have also shown promise in developing countries, although evidence is still scarce [5-11] and largely not derived from randomized trials. Special considerations apply to P4P programs in developing countries. Sustainability is of particular importance in resource-constrained settings where a large portion of the health budget may be derived from donor funding. The role of the donor in setting priorities and criteria for performance may undermine sustainability once donor funding is exhausted. This is critical because participant improved behavior has been shown to reverse when incentives are withdrawn [12]. Overall, there is a lack of methodologically rigorous evaluation of P4P approaches, particularly from the developing world.

Malaria case management in public health facilities is an example of systemically poor provider behavior that may be amenable to change using P4P incentives. Over-treatment of fevers with antimalarials is a significant problem in malaria-endemic areas with serious consequences. In a typical rural health facility in Kenya, more than 90% of patients with fever are prescribed an antimalarial drug when no diagnostic tests are available [13]. Even when diagnostic tests are available, 40% to 80% of patients with a negative test are nonetheless prescribed antimalarials [14-19]. Inappropriate treatment of non-malaria fevers with antimalarials often results in failure to treat the true cause of the fever and may be life-threatening for the patient. Current first-line therapies, moreover, cost 10 to 20 times more than their predecessors. Overuse of new, expensive antimalarial drugs puts a financial strain on the government health system and jeopardizes the sustainability of donor-subsidized drug procurement programs. It can also accelerate the establishment and spread of drug resistance and reduce the useful therapeutic life of the drugs.

Provider training alone has failed to reduce the proportion of non-malaria fevers treated with antimalarials [20,21]. Over-treatment of fevers with antimalarials is partly due to prescriber habits, but is also due to poor institutional incentives to improve prescribing and diagnostic testing practices. In Kenya as in other sub-Saharan African countries, neither the government, the health facility, nor the patient pays for antimalarials, but a fee is charged to the patient for the diagnostic test. This creates a perverse incentive against laboratory confirmation of malaria.

We are testing a novel strategy to introduce financial considerations into decision making at the level of the facility through facility-based incentives in order to increase diagnostic testing, improve appropriateness of prescription practices, and increase adherence to the results of diagnostic tests for malaria. Our approach differs from previous P4P programs in several important ways: previous P4P strategies in Africa emphasized volume of patients receiving services whereas our incentives are applied around quality of case management; our incentives are designed to be cost-saving and therefore sustainable; and our incentives are not applied to individual provider salaries, but are facility-based incentives, thus allowing money to be reinvested into the health system. Facility-based incentives emphasize the teamwork across departments (laboratory, pharmacy, records, and clinical services) that is required for good case management.

There are several key contextual factors that make this approach feasible and timely in Kenya. First, the recently established Health Sector Services Fund (HSSF) provides a small amount of money directly to peripheral government health facilities on a quarterly basis to support local priorities and activities. This new initiative included training on budgeting, accounting, and procurement. It provides an avenue through which incentives can be channeled and monitored. Because the HSSF is nationwide, it also provides a mechanism by which a successful incentive scheme could be quickly and effectively scaled up. Second, changes in levels of funding to international health programs such as the Global Fund and President’s Malaria Initiative is generating concern about the future sustainability of donor-funded health programs. After a rapid expansion of malaria control interventions between 2004 and 2009, global funding for malaria prevention and control leveled off between 2010 and 2012 and delivery of some life saving commodities has slowed [22]. Initiatives that contribute to reducing cost while improving quality are urgently required. Finally, the activities being incentivized are not new activities, rather we are incentivizing adherence to evidence-based guidelines that are already formalized in government policy.

We describe the design of a cluster-randomized controlled study to investigate the role of sustainable institutional incentives to improve management of malaria in peripheral health facilities. This study will demonstrate whether facility-based rather than individual incentives are compelling enough to change provider behavior and whether these incentives lead to cost savings as a result of targeted drug consumption.

## Methods/design

This is a cluster-randomized trial with 10 health centers enrolled in the intervention arm and eight health centers enrolled in the comparison arm.

### Malaria diagnosis and treatment in Kenya

In 2009, Kenya reported nine million cases of malaria (clinically diagnosed and laboratory-confirmed), but dispensed 20 million treatment courses of artemether lumefantrine (AL), the first-line antimalarial treatment for uncomplicated malaria. This represents approximately 10 million USD wasted each year. Although malaria has historically been the leading cause of morbidity and mortality among children in sub-Saharan Africa, successful malaria control programs have succeeded in reducing malaria-related admissions and deaths in many countries, including Kenya [23-26]. As malaria declines, a greater fraction of febrile episodes are due to other causes. The World Health Organization (WHO) and the Kenya Division of Malaria Control now recommend diagnostic testing before prescription of antimalarials whenever possible [27,28]. In spite of this, a large proportion of non-malarial fevers continue to be treated as malaria. In a typical rural health facility, more than 90% of patients are prescribed an antimalarial drug when no diagnostic tests are available [13]. Even when diagnostic tests are available, 40% to 80% of patients with a negative test are nonetheless prescribed antimalarials [14-19].

### Study setting

This study is being conducted in Western and Rift Valley Provinces in western Kenya. Malaria endemicity is high in Western Province, particularly near Lake Victoria. However, Rift Valley Province has low transmission and is prone to epidemics when climate conditions are suitable.

The large majority of Kenyans access healthcare through a network of government health facilities. Dispensaries and health centers are the most basic health facilities and are distributed throughout the country. These facilities are staffed by nurses and clinical officers and offer ambulatory care and preventive services. More complicated cases or those requiring hospitalization are referred to district hospitals. Health centers usually have basic laboratory services and a laboratory technologist on staff.

### Policy context and collaboration with the government of Kenya

The Ministry of Public Health and Sanitation through the Division of Malaria Control (DOMC) was engaged from the project conception and design stage, even before applying for funding. This was done to ensure that project goals aligned with government priorities for malaria control. DOMC is represented on our study advisory committee and among the investigative team. Approval for the study was also sought and granted from the Division of Health Facility Services that oversees HSSF. district-level Ministry representatives are involved in all routine visits to facilities.

This avenue of investigation aligns with government priorities for developing future programs and policies. The HSSF program was designed to incorporate a P4P component. This is the first experiment of a P4P scheme within the HSSF program. It is critical for the Ministry of Health to understand the potential and possible limitations of institutional (rather than personal) incentives in improving care.

International policies for support to malaria-endemic countries are also changing. Global Fund is considering changing drug procurement policies so that recipient countries must budget for drug procurement for public and private drug subsidies from their grants [29]. Rather than having specific funds allocated to drug procurement, they will have a total resource envelope from which they must carve their drug budget. Targeting antimalarials to those who have been confirmed to have malaria will greatly reduce drug expenditures, allowing funds to be channeled to diagnostics or prevention.

### Facility selection

Eighteen health centers from seven districts were selected for participation. After permission for the study was obtained from the national and provincial health leadership, health facilities that met the inclusion criteria were assembled into a sampling frame. Health centers within western and the northern half of Rift Valley provinces were eligible for the study if they currently had capacity to diagnose malaria by microscopy. There were 61 eligible health centers. Eighteen health centers were chosen by simple random sampling. These facilities were within eight districts in two provinces. Two districts had only one facility selected, so one district was dropped and a facility added in the second to ensure that each district had at least two facilities. The districts were contacted to confirm the facilities met the inclusion criteria (government-owned with a functional laboratory and staff). Two facilities were found to not meet the inclusion criteria. These facilities were replaced with eligible facilities within the same district.

### Training

One laboratory technician from each facility was sent to a two-week intensive training program for microscopic diagnosis of malaria at the Malaria Diagnostic Center in Kisumu, Kenya. This training has been shown to improve sensitivity and specificity of microscopic diagnosis of malaria to >85% and 90%, respectively [30]. The trainees participating from study facilities improved both sensitivity and specificity an average of 18 percentage points during the training. Two clinicians from each facility, as well as their district-level supervisors (the District Clinical Officer and District Public Health Nurse), were invited to attend a two-day clinical training to review basic management of childhood fevers, including an in-depth review of the National Guidelines for the Diagnosis, Treatment, and Prevention of Malaria in Kenya, (2010). Forty percent had never received formal training on the revised guidelines, which included new criteria for testing for malaria before prescribing antimalarials.

### Randomization

After training and baseline assessments were complete, the facilities were randomized by district. Randomization was performed in an open meeting with representatives from each facility present. Red and black chips were drawn to determine the composition of each arm. For districts with two participating facilities, one red and one black chip were put into a bin. A representative from each facility drew a chip. For districts with four participating facilities, two black and two red chips were put in the bin. For districts with three facilities, two red and two black chips were put in the bin to ensure each facility had an equal chance of being in the intervention arm.

### Performance-based incentives

The intervention we are testing is the use of facility-level, performance-based incentives to improve quality of care for suspected malaria fevers. All eighteen facilities will receive monthly external quality assurance (EQA) of their laboratory malaria diagnosis. A random sample of 15 positive slides and 15 negative slides will be selected from each facility each month and re-read by an expert microscopist. The microscopist is blinded to the facility results. Sensitivity and specificity of clinical microscopy is calculated with reference to the expert results.

The ten facilities enrolled in the intervention arm will receive a financial incentive that is calculated based on their diagnosis and prescription practices for malaria over that quarter. The incentives will be provided in the form of additional resources (top-up) to the facility to augment their Health Sector Services Funds. The incentive money is subject to the same restrictions as the HSSF; it can only be used for facility needs, including equipment, supplies, repairs, and basic labor [31].

The incentive scheme is designed to maximize the effect of the incentives by close temporal relationship between the desirable behavior and the reward. Each facility has the possibility of earning the maximum amount of incentive payments based solely on their performance rather than on a zero-sum game where high performers are rewarded and low performers are penalized [32]. None of our incentives are punitive, and we designed the incentives to foster cooperation between all staff members to achieve the targets.

Incentives will be calculated on a quarterly basis according to a set of indicators agreed on by the participating districts and the investigators (Table 1). The indicators include basic recordkeeping, quality of laboratory diagnosis, and clinician adherence to the laboratory diagnosis. The intervention will last 12 months. Each indicator is weighted according to importance (record keeping indicators are worth only 5% each of the total, for example). Each quarter, a facility can earn 25% to 100% of the total value of an indicator depending on their performance. Optimal performance could return as much as Ksh100,000 ($1,176 UDS) per quarter to each facility.

**Table 1:** Indicators for calculating incentives

	**Weight**	**Scale**	Value in KES*	**Value in US$**
Percent of laboratory registry entries with patient number	5%	100% - full marks	5,000	59
		90% - 60/100	3,000	35
		85% - 50/100	2,500	29
		< 85 – 0/100	0	0
Percent of artemether-lumefantrine registry entries with patient number	2.5%	100% - full marks	2,500	29
		90% - 60/100	1,500	18
		85% - 50/100	1,250	15
		< 85 – 0/100	0	0
Percent of antibiotics registry entries with patient number	2.5%	100% - full marks	2,500	29
		90% - 60/100	1,500	18
		85% - 50/100	1,250	15
		< 85 – 0/100	0	0
Sum of sensitivity and specificityof malaria microscopy	20%	190+ − full marks	20,000	235
		170+ − 80/100	16,000	188
		150+ − 60/100	12,000	141
		< 150 – 0/100	0	0
Percent of patients given antimalarials without a diagnostic test	30%	< 5% - full marks	30,000	353
		5-10% - 70/100	21,000	247
		11-20% - 50/100	15,000	176
		>20% - 0	0	0
Percent of patients with a positive test given antimalarials	15%	100% - full marks	15,000	176
		90% - 70/100	10,500	124
		80% - 50/100	7,500	88
		70% - 25/100	3,750	44
		< 70 – 0/100	0	0
Percent of patients with a negative test given antimalarials	25%	< 5% - full marks	25,000	294
		5-10% - 70/100	17,500	206
		11-20% - 50/100	12,500	147
		>20% - 0	0	0
TOTAL	100%		100,000	1,176

*Approximately 85 Kenya shillings is equal to one US dollar.

### Data collection

Data collection began in August 2012. August to October 2012 is the post-training, pre-intervention baseline period. Data collected from October 2012 through September 2013 will describe the effect of the intervention. Each month, a sample of slides is collected at each facility and re-read by the study microscopist. The selected slides are matched to the prescription forms and outpatient registers to determine whether the patient was appropriately prescribed AL or alternative therapies and to record patient information such as age, gender, and date of visit. All data are collected from standard government-issued facility register books on a monthly basis.

### Sample size and primary endpoint

We calculated sample size and power based on a cluster-randomized, difference-of-proportions test with the primary outcome being the proportion of slide-negative children receiving antimalarials at one year [33]. Based on data from a representative facility, we anticipate that each facility sees approximately 300 sick children per month, at least 30% of these receive a malaria test, and 50% to 90% test negatively. Based on previous studies [19], we conservatively estimate that 50% of slide-negative children in the control arm will receive antimalarials. From these data, we calculate that 18 facilities (nine per arm) with 26 slide-negative children per facility will achieve 90% power to detect a minimal clinically relevant difference of 0.15 in the proportion of negative children receiving antimalarials, assuming a two-sided test at the alpha = 0.05 significance level and an intra-cluster correlation (ICC) of 0.002.

Secondary outcomes include the odds of being prescribed AL without a malaria diagnostic test and the odds of being prescribed AL if the diagnostic test is positive. The sensitivity and specificity of the clinical microscopy compared to the expert study microscopist will be monitored continuously. The frequency and duration of stockouts are being monitored to determine whether more rational use of antimalarials reduces drug shortages. We are also recording alternative therapies prescribed to children with negative blood smears, in particular changes in prescription of other antimicrobials.

### Funding and ethical review

This study is being funded by the National Institute of Allergy and Infectious Diseases at the National Institutes of Health. The protocol was reviewed and approved by Duke University Institutional Review Board and Moi University Institutional Research and Ethics Committee (IREC/2012/18 Approval # 000804).

### Trial status

#### Baseline assessment

Baseline assessments were carried out in June and July 2012. Most facilities had at least one clinical officer and between three to nine nurses, but only three facilities had a trained pharmacy technologist to dispense medicines. None of the facilities had a physician on staff, as is the norm for health centers and dispensaries in Kenya. All facilities reported they were regularly receiving HSSF and used the money for supplies, paying unskilled labor (janitors, records clerks, gardeners, etc.), transporting patients who needed to be referred, and paying utility bills (water and electricity).

All facilities reported frequent problems with shortages of antimalarials and antibiotics. In five facilities, no AL was available on the day of the assessment and none had been provided in the last 6 to 12 months. In one facility, no antibiotics were available on the day of the visit. In other facilities, between two and five different antibiotics were in stock (erythromycin, injectable penicillin, septrin, doxycycline, amoxicillin). All facilities provided drugs free of charge to patients less than five years of age, in accordance with government policy. Four facilities charged a nominal fee for antimalarials to patients older than five years and five charged a nominal fee for antibiotics for older patients.

The percent of reported malaria cases that were confirmed by parasitological diagnosis varied widely between facilities and across age groups (Table 2). In some facilities, only a small percentage of children under five years who received a diagnosis of malaria were sent for parasitological confirmation. In one facility, 66% of reported cases in young children were laboratory-confirmed. In general, the facilities located in Western Province where malaria transmission is high reported a significantly higher percentage of laboratory-confirmed cases than their counterparts in Rift Valley where malaria transmission is low and epidemic-prone. Patients older than five years were slightly less likely to have a laboratory-confirmed diagnosis than young children.

**Table 2:** Malaria cases management in enrolled facilities prior to training or implementation of incentives

	**Over 5 years**				**Under 5 years**				**Summary**			
**Facility**	**Clinical**	**Confirmed**	**% conf.**	Cost (KES*)	**Clinical**	**Confirmed**	**% conf.**	Cost (KES*)	**Total clinical and confirmed (all ages)**	**Total AL dispensed**	**Total slides read (Jan-Mar)**	**Percent positive**
**Comparison**												
Comparison 1	431	2	0%	0	267	1	0%	0	701	1,190	110	1
Comparison 2	427	134	24%	50	362	129	26%	0	1,052	1,410	730	37
Comparison 3	317	360	53%	30	459	544	54%	0	1,680	1,095	1,961	27
Comparison 4	635	197	24%	50	554	198	26%	30	1,584	1,466	850	24
Comparison 5	856	100	10%	40	988	123	11%	0	2,067	4,406	934	28
Comparison 6	534	517	49%	20	427	453	51%	0	1,931	O/S	2,434	28
Comparison 7	547	58	10%	50	311	4	1%	0	920	7,098	135	27
Comparison 9	639	238	27%	20	406	160	28%	0	1,443	O/S	1,510	23
**Intervention**												
Intervention 1	307	53	15%	50	113	10	8%	0	483	331	229	19
Intervention 2	270	81	23%	20	159	19	11%	0	529	O/S	266	10
Intervention 3	325	0	0%	50	919	0	0%	0	1,244	1,146	-	-
Intervention 4	133	434	77%	20	191	389	67%	20	1,147	1,545	647	57
Intervention 5	91	369	80%	30	59	391	87%	20	910	1,183	709	58
Intervention 6	1,289	6	0%	40	1,168	7	1%	0	2,470	1,910	0**	N/A
Intervention 7	1,110	64	5%	50	842	22	3%	0	2,038	2,173	-	-
Intervention 8	577	188	25%	20	265	405	60%	0	1,435	1,758	949	38
Intervention 9	367	155	30%	20	161	70	30%	0	753	O/S	1,781	22
Intervention 10	457	110	19%	30	211	57	21%	0	835	-	-	-

Confirmed cases are those who received a diagnostic test. All figures are the total for January through March 2012.

*Approximately 85 Kenya shillings is equal to one US dollar.

**Intervention facility 6 did not have a laboratory technician until May 2012.

O/S = out of stock of AL during Jan-March 2012.

- = Data not available.

Nearly all facilities charged a fee for laboratory testing of patients over five years, but only three charged for malaria diagnosis in children under five. There was no clear trend in percent of cases confirmed and price of a test. However, there was an observed trend in the percentage of cases confirmed and total number of cases. The percentage of cases confirmed was markedly lower amongst facilities who reported >800 total malaria cases in the first quarter of 2012.

The number of antimalarial prescriptions dispensed exceeded the total number of reported malaria cases (clinical plus confirmed) in 70% of facilities that did not experience stock-outs. In one facility, the number of antimalarial prescriptions dispensed was more than 6,000 treatment courses in excess of the total malaria cases.

## Discussion

Here we describe a study to test a novel strategy to introduce financial considerations into decision making at the level of the facility in order to increase diagnostic testing, improve prescription practices, and increase adherence to the results of diagnostic tests. Personal incentive schemes to healthcare providers have been shown to improve patient volume and uptake of interventions in developing countries [5,7,8]. In most reports, the measures of performance did not include technical quality, and there is a lack of methodologically rigorous evaluations of incentive programs, either personal or institutional, in the healthcare setting in the developing world [6,9]. Furthermore, there is concern that incentives may not be sustainable without external funding, particularly in the public sector [10]. Our study focuses on a quality indicator and will use an experimental design to measure the impact of incentives provided at the facility level. The incentives offered are designed to be sustainable by reducing wastage of expensive antimalarials.

Over the last decade, investment in malaria control in has significantly improved coverage of both preventive interventions and effective treatment leading to marked reductions in malaria [24-26]. As malaria transmission declines, a greater fraction of pediatric fevers are due to other causes. As a result, guidelines issued by the WHO and the Government of Kenya both mandate diagnostic testing before treatment with antimalarials. However, treatment and prescription practices have not adapted to changes in malaria burden or treatment guidelines. Despite improved access to malaria diagnosis, a large fraction of non-malaria fevers are treated with antimalarial drugs. For example, in one rural facility in our study area, outpatient records showed that in 2003, 66% of patient with a negative malaria test were given an antimalarial [34]. In the same facility eight years later, the proportion of positive blood smears has declined from 13% to less than 3%, but the percentage of all patients treated with an antimalarial has risen from 12% to 23% (O’Meara, personal communication). In six months, 670 patients had a blood smear examined for parasites, with parasites detected in only 19 smears; yet, 2,100 patients were given an antimalarial. This means that out of 2,100 fevers treated with malaria drugs, a staggering 97% of fevers were potentially incorrectly treated, and upwards of 2,000 USD of unnecessary drugs dispensed in one facility over six months.

Overuse of antimalarial drugs places an enormous financial strain on limited health sector budgets, taxing both health systems and donor resources. Overuse of drugs is a significant impediment to sustainability of international investments in the fight against malaria. Current first line drugs such as AL cost 10 to 20 times more than their predecessors. Kenya spends between 10 and 12 million USD per year on procuring antimalarial drugs alone, even though prices are highly subsidized by the international donor community. It has been estimated that the cost of outpatient treatment of fevers could be reduced by more than 50% if antimalarial treatment was restricted to those with a positive test [35,36].

Research indicates that simply providing training in clinical management of fevers and the use of malaria diagnostics does not correct the problem of over-prescription of antimalarial drugs and inappropriate treatment of non-malaria fevers [21]. Drug consumption and diagnostic practices are driven by financial pressures. One problem with the current approach is that the financial incentives for diagnostic testing and correct prescribing practices are not applied at the levels where these decisions are actually made. In Kenya, drugs are provided free to government health facilities, which in turn provide the drugs for free to patients. Thus there is no financial incentive for the facility, the individual prescribers, or the patient to consider the cost of that drug or the implications of overuse for drug resistance. In contrast, if microscopic testing is available, the patient is charged approximately US $0.75 for a malaria diagnostic test. Thus the current situation at the facility level makes it less likely that patients will be tested, and more likely that they will be prescribed antimalarials regardless of their parasitological status.

Under our incentive program, health centers earn payments based on improving the quality of their laboratory diagnosis of malaria, increasing the percentage of patients who are tested before being prescribed antimalarials, and reducing the percentage of patients with a negative test who receive antimalarials. The financial incentives offered in this study are cost-effective because they reduce drug wastage and could provide cost savings to the government. The size of the incentive is based on the cost savings associated with reducing inappropriate drug use. The proposed facility-level incentive could be scaled up nationally through decentralized facility funding being rolled out nationwide in Kenya, as well as in other malaria endemic countries.

As with all P4P programs, there is valid concern about ‘gaming’ the system to improve apparent performance and maximize payouts (for a tongue-in-cheek guide to gaming, see [37]). We cannot rule out the possibility of gaming, however, the indicators being evaluated cut across several departments and the degree of collusion required to cheat may be prohibitive. Furthermore, there are no individual-based payouts that may reduce the attractiveness of ‘gaming’. This is a double-edged sword—it remains to be seen whether incentive schemes without a personal benefit will motivate change in behavior. One significant limitation of our study is that it focuses narrowly on malaria case management. There is a concern that emphasis on malaria case management in response to the incentives offered may adversely affect the quality of other services. This remains to be seen and we are following non-incentivized indicators in order to quantify possible negative impact.

This study will advance our understanding of how to overcome obstacles to successful implementation of the current clinical care guidelines for malaria, as well as contribute to the evidence-base regarding P4P in the context of a developing country health system.

## Competing interests

Authors declare that they have no competing interests.

## Authors’ contributions

DM, JL, IM, WPO contributed to study design, drafting of the protocol and implementation of the study. JA contributed to study design and designed the training components. BN contributed to study design and data collection tools. WPO wrote the initial draft of the manuscript which received critical review, editing, and approval by all co-authors. All authors read and approved the final manuscript.

## Funding

This study is funded by an Dissemination and Implementation Science award to W. Prudhomme-O’Meara from the National Institutes of Allergy and Infectious Disease of the National Institutes of Health (1R21AI095979-01A1).
